# An analysis of the effect of statins on the risk of Non‐Hodgkin's Lymphoma in the Women’s Health Initiative cohort

**DOI:** 10.1002/cam4.1368

**Published:** 2018-04-02

**Authors:** Pinkal Desai, Robert Wallace, Matthew L. Anderson, Barbara V. Howard, Roberta Ray, Chunyuan Wu, Monika Safford, Lisa W. Martin, Nicolas Schlecht, Simin Liu, Dominic Cirillo, Allison Jay, JoAnn E. Manson, Michael S. Simon

**Affiliations:** ^1^ Weill Cornell Medical College New York New York; ^2^ Department of Epidemiology University of Iowa College of Public Health Iowa City Iowa; ^3^ Baylor College of Medicine Houston Texas; ^4^ MedStar Health Research Institute and Georgetown/Howard Universities Center for Clinical and Translational Science Washington District of Columbia; ^5^ Fred Hutchinson Cancer Research Center Seattle Washington; ^6^ George Washington University Washington District of Columbia; ^7^ Albert Einstein College of Medicine Bronx New York; ^8^ Brown University School of Public Health Providence Rhode Island; ^9^ St John Providence Hospital Warren Michigan; ^10^ Brigham and Women's Hospital and Harvard Medical School Boston Massachusetts; ^11^ Department of Oncology Karmanos Cancer Institute Wayne State University Detroit Michigan

**Keywords:** NHL, prevention, Statins

## Abstract

Statins have been shown to induce a phosphoprotein signature that modifies MYC (myelocytomatosis viral oncogene) activation and to have anti‐inflammatory activity that may impact the risk of Non‐Hodgkin's lymphoma (NHL). We analyzed the relationship between statins and risk of NHL using data from the Women's Health Initiative (WHI). The study population included 161,563 postmenopausal women ages 50–79 years from which 712 cases of NHL were diagnosed after 10.8 years of follow‐up. Information on statin use and other risk factors was collected by self‐ and interviewer‐administered questionnaires. Multivariable‐adjusted HR and 95% CI evaluating the relationship between statin use at baseline, as well as in a time‐dependent manner and risk of NHL, were computed from Cox proportional hazards analyses. A separate analysis was performed for individual NHL subtypes: diffuse large B‐Cell lymphoma (DLBCL) (*n* = 228), follicular lymphoma (*n* = 169), and small lymphocytic lymphoma (*n* = 74). All statistical tests were two‐sided. There was no significant association between use of statins at baseline and risk of NHL (HR 0.85, 95% C.I. 0.67–1.08). However, in the multivariable‐adjusted time‐dependent models, statin use was associated with a borderline lower risk of NHL (HR 0.81, 95% C.I. 0.66–1.00). Considering subtypes of NHL, statin use was associated with a lower risk of DLBCL (HR 0.62, 95% C.I. 0.42–0.91). This effect was driven by lipophilic statins (HR 0.62, 95% C.I. 0.40–0.96). In the WHI, statins were associated with a lower overall risk of DLBCL, particularly attributable to lipophilic statins. These results may have impact on primary or secondary prevention of NHL, particularly DLBCL.

## Introduction

Statins are the most widely prescribed cholesterol‐lowering drugs in the United States. According to the latest Centers for Disease Control data in the years 2005–2008, the percent of US adults taking statins had increased to 25% 1 compared to an estimated 11.7% in 2003–2004 2. Statins are a class of drugs used for lowering cholesterol and act by competitive inhibition of hydroxy methyl glutaryl coenzyme A (HMG CoA), which is the rate‐limiting enzyme in the mevalonate pathway. Inhibition of the mevalonate pathway also leads to lower levels of downstream products including farnesyl diphosphate (FPP), geranylgeranyl diphosphate (GGPP), and dolichol 3, 4 which may have implications for chemoprevention of cancer.

Mouse models have demonstrated proapoptotic and antitumor activity of statins against lymphoma 5. Statins have also been shown to prevent and reverse MYC‐induced lymphoma 6. In addition, the anti‐inflammatory effect of statins may be an important mechanism of reduction in risk of lymphoma as chronic inflammation may be a risk factor 7.

Epidemiological studies of the association of statins and risk of lymphoid malignancies have shown mixed results with some studies showing lower risk 8, 9, 10 while others showing increased risk or no association 11, 12. Statin use and its association with solid malignancies has been extensively studied in the Womens Health Initiative cohort 13, 14, 15. In this report, we analyze the relationship between statin use, statin type, potency, and lipophilicity with risk of NHL overall and NHL subtypes in a large, multisite cohort of postmenopausal women.

## Methods

### Study population

The study population included 161,808 postmenopausal women aged 50 to 79 enrolled in the Women's Health Initiative (WHI) Clinical Trial (CT) and Observational Study (OS) from October 1, 1993 to December 31, 1998. Study implementation details have been published previously 16, 17, 18. Follow‐up continued from study initiation until planned termination in March, 2005, and thereafter for participants providing reconsent. The data collection for the current analysis was updated through September 2012. Some women continue to be followed in the extension study. We excluded women from the analysis who had a prior history of lymphoma (*n* = 243) and for whom there was no information on statin use (*n* = 2) for a final analysis cohort of 161,563.

### Statin exposure

Statin use was defined as use of any HMG CoA reductase inhibitor. Statins were classified as lipophilic (lovastatin, simvastatin, fluvastatin, atorvastatin) or hydrophilic (pravastatin) and by potency as low potency (fluvastatin and lovastatin), medium potency (pravastatin), and high potency (simvastatin and atorvastatin) 19, 20.

Information on statin use was collected at baseline in the WHI, and thereafter follow‐up information on statin use was determined at year 3 in the OS participants and years 1, 3, 6, and 9 in the CT participants. At baseline and at each follow‐up visit, participants were asked to bring all of their current prescription medications to the clinic visit (or first interview at baseline). At those visits, interviewers entered each medication name directly from the medication containers into the WHI database, which assigned drug codes using Medispan software (First DataBank, Inc., San Bruno, CA). At the time of the visit, women also reported duration of statin use.

### NHL

Self‐report of NHL was locally verified at each clinic by medical record and pathology report review by centrally trained WHI physician adjudicators. Central adjudication and coding of histology were performed at the Clinical Coordinating Center using the Surveillance, Epidemiology, and End Results Program (SEER) coding system. Only NHL cases confirmed by central adjudication were included in the analysis (1091 cases). Information on subtype of NHL was available and included 368 cases of diffuse large B‐Cell lymphoma (DLBCL), 255 cases of follicular lymphoma, 96 cases of small lymphocytic lymphoma (SLL), and the rest were categorized as other lymphoma type. There were two cases where the diagnosis was not adjudicated and were not included in the analysis.

### Covariates

Information on all covariates was collected at study entry including sociodemographic characteristics, clinical history as well as factors associated with healthcare utilization which might impact both statin utilization and detection or diagnosis of NHL.

### Statistical analysis

The characteristics of statin users at baseline were compared with those of nonusers by chi‐squared tests. Annualized rates of NHL (incidence per person‐year) were calculated according to the use of statins. We performed selected subgroup analyses by information on duration of statin use (only available at baseline; <1 year, 1–<3 years, and ≥3 years), statin type, potency, and lipophilic status. Women who reported using two or more statins were included in analyses that compared statin use to none, but were excluded from analyses that examined details of statin use (by type, potency, or lipophilic status). Hazard ratios (HRs) for NHL among statin users versus nonusers, and 95% confidence intervals (CIs) were computed from Cox proportional hazards analyses. A Cox model that included statin use and the interaction of statin use with follow‐up time and testing for a zero coefficient on the interaction term was used to conduct tests for the proportional hazards assumptions.

While only 7.5% of WHI participants used statins at baseline, approximately 25% were using statins at the end of the trial completion. In order to account for increasing statin use during follow‐up, we examined the association of statin use and NHL using time‐dependent analyses. To evaluate the effect of change in statin use overtime, final models were run by entering statin use as a time‐dependent exposure and using updated information on statin use gathered at each follow‐up time point. Cases of NHL as well as noncases were censored if they occurred more than 3 years after last medication update in the OS participants to closely parallel the follow‐up experience of women in the CT. A set of covariates was selected a priori for adjustment of potential confounding based on covariates that are associated with risk of NHL or heathcare utilization. These included age (continuous), history of lupus and rheumatoid arthritis at WHI baseline (yes/no), and current medical care provider (at WHI baseline, yes/no). Additionally, all models included strata for age group at WHI enrollment, extension study (yes/no), and study group/trial participation.

Additional WHI baseline variables were individually tested as possible confounders: BMI, race/ethnicity, smoking status, education, percent energy from fats, recreational physical activity, waist circumference, aspirin use, history of cardiovascular diseases, and postmenopausal hormone use. We compared results from models that included all of the a priori covariates and each potential confounder to models including the a priori covariates only. Covariates were considered to be confounders if their inclusion in a model resulted in a change in any of the HRs for the statin variables by 10% or more. None of the additional variables met these criteria; therefore, the results presented are those from the models adjusted for the a priori covariates.

Separate analyses were conducted for individual subtypes of NHL (diffuse large B‐cell lymphoma (DLBCL), small lymphocytic lymphoma (SLL), marginal zone lymphoma (MZL), and follicular lymphoma).

## Results

Table 1 shows the baseline characteristics of statin users and nonusers. Statin users were more likely to be older, have a higher BMI and waist circumference, report having a healthcare provider, and to have more than 30% intake of energy from fat.

**Table 1:** Baseline characteristics by statin use

	No		Yes		
	*N*	%	*N*	%	*P*‐value
Age group at screening					
50–59	51,368	34.35	2189	17.88	<.0001
60–69	66,219	44.27	6370	52.03	
70–79	31,976	21.38	3684	30.09	
Median age at screening	63 year		66 year		<.0001
Race/ethnicity					
White	12,3494	82.57	10046	82.06	<.0001
Black	13,499	9.03	1118	9.13	
Hispanic	6100	4.08	384	3.14	
American Indian	665	0.44	48	0.39	
Asian/Pacific Islander	3720	2.49	470	3.84	
Unknown	2085	1.39	177	1.45	
Education					
<HS diploma/GED	7850	5.29	794	6.53	<.0001
HS diploma/GED	25,061	16.88	2563	21.07	
>HS diploma/GED	115,517	77.83	8805	72.40	
Smoking status					
Never	75,545	51.18	5884	48.78	<.0001
Past	61,667	41.77	5442	45.12	
Current	10,406	7.05	736	6.10	
Alcohol					
Non drinker	43,579	29.36	4221	34.70	<.0001
≤1 drink/day	48,774	32.86	4088	33.60	
>1 drink/day	56,064	37.77	3856	31.70	
Hormone therapy use					
Never	65,272	43.68	5612	45.89	<.0001
Past	23,760	15.90	2169	17.74	
Current	28,218	40.42	2222	36.37	
Body mass index (kg/m^2^)					
<25	53,313	35.96	3021	24.88	<.0001
25–<30	50,858	34.31	4822	39.72	
≥30	44,068	29.73	4297	35.40	
Median body mass index (kg/m^2^)	26.8		28.0		<.0001
Physical activity, met/wk					
Inactive 0 METs	22,703	15.94	1759	14.73	<.0001
[0,3.75) METs	20,789	14.60	1831	15.33	
[3.75, 8.75) METs	29,140	20.46	2626	21.99	
[8.75, 17.5) METS	32,101	22.54	2784	23.31	
≥17.5 METS	37,660	26.45	2942	24.64	
Waist circumference >88 cm					
No	91,023	61.08	6153	50.43	<.0001
Yes	58,000	39/92	6049	49.57	
≥30% energy from fat					
No	52,486	35.16	5370	43.92	<.0001
Yes	96,800	64.84	6856	56.08	
Current healthcare provider					
No	9800	6.62	195	1.61	<.0001
Yes	138,286	93.38	11950	98.39	
History of lupus					
No	146,729	99.50	12,133	99.59	0.1587
Yes	743	0.50	50	0.41	
History of rheumatoid arthritis					
No arthritis	78,683	54.39	5484	45.99	<.0001
Rheumatoid arthritis	7185	4.97	686	5.75	
Other arthritis/don't know	58,797	40.64	5754	48.26	

Table 2 shows the distribution of statin use at baseline by type, duration, potency, and lipophilicity. Simvastatin was the most common statin used with 29.3% of participants using it at baseline. The majority of statin users took lipophilic statins (69.4%) and 39.3% of users were on a statin classified as low potency, 38.2% high potency and 22.5% medium potency. Among statin users at baseline, the percentage of participants using statins for <1 year, 1–<3 years, and ≥=3 years was 33.1%, 33.9%, and 32.9%, respectively.

**Table 2:** Distribution of statins by type, duration, and other statin characteristics

	No		Yes	
	*N*	%	*N*	%
Statin type				
No statin use	14,9563	100.00		
Atorvastatin calcium			961	7.85
Fluvastatin sodium			1484	12.12
Lovastatin			3204	26.17
Pravastatin sodium			2686	21.94
Simvastatin			3589	29.31
Two or more statins			319	2.61
Statin potency				
No statin use	149,563	100.00		
Low (Lovastatin, Fluvastatin)			4688	39.32
Medium (Pravastatin)			2686	22.53
High (Simvastatin, Atorvastatin)			4550	38.16
Lipophilicity				
No statin use	149,563	100.00		
Lipophilic statins			8277	69.41
Hydrophilic statins (pravastatin)			3647	30.59
Statin use in years				
No statin use	149,563	100.00		
<1			4054	33.11
1–<3			4151	33.91
≥3			4038	32.98

Table 3 shows the relationship between baseline statin use and risk of NHL by overall statin use, statin type, potency, and lipophilicity. The annualized rate of NHL among statin users and nonusers was 0.06% and 0.05%, respectively, with a mean follow‐up of 12.57 years for statin users and 11.82 years for nonusers. Overall statin use at baseline was not associated with risk of NHL (HR 0.85, 95% C.I. 0.67–1.08). There was no significant association between statin lipophilicity, potency, type or duration of use, and risk of NHL.

**Table 3:** Non‐Hodgkin's lymphoma (NHL) incidence (annualized %) and HRs by statin use

					Age adjusted				Multivariable adjusted			
	*N*	NHL	Annualized %	Mean fup (year)	Hazard ratio	Lower CL	Upper CL	*P*‐value	Hazard ratio	Lower CL	Upper CL	*P*‐value
Statin use												
No	14,9338	1011	0.05	12.57	1.00			0.38	1.00			0.18
Yes	12,225	80	0.06	11.82	0.90	0.72	1.14		0.85	0.67	1.08	
Statin type												
No statin use	14,9338	1011	0.05	12.57	1.00				1.00			
Atorvastatin	957	3	0.03	10.85	0.45	0.14	1.39	0.16	0.45	0.14	1.40	0.17
Fluvastatin	1484	8	0.05	11.59	0.75	0.37	1.50	0.41	0.65	0.31	1.38	0.26
Lovastatin	3197	24	0.06	12.25	1.01	0.67	1.52	0.96	0.91	0.59	1.40	0.67
Pravastatin	2683	20	0.06	11.80	1.03	0.66	1.60	0.90	0.94	0.59	1.51	0.81
Simvastatin	3585	22	0.05	11.82	0.86	0.56	1.31	0.47	0.87	0.57	1.32	0.50
Lipophilicity												
No statin use	14,9338	1011	0.05	12.57	1.00				1.00			
Lipophilic	9223	57	0.05	11.83	0.85	0.65	1.11	0.24	0.80	0.61	1.06	0.13
Hydrophilic	2683	20	0.06	11.80	1.03	0.66	1.60	0.90	0.94	0.59	1.51	0.81
Statin potency												
No statin use	14,9338	1011	0.05	12.57	1.00				1.00			
Low (Lovastatin, Fluvastatin)	4681	32	0.06	12.04	0.93	0.65	1.32	0.68	0.83	0.57	1.21	0.33
Medium (Pravastatin)	2683	20	0.06	11.80	1.03	0.66	1.60	0.90	0.94	0.59	1.51	0.81
High (Simvastatin, Atorvastatin)	4542	25	0.05	11.62	0.77	0.52	1.15	0.20	0.78	0.52	1.16	0.22
Statin duration												
0	14,9338	1011	0.05	12.57	1.00				1.00			
1–<3 year	4023	28	0.06	11.90	0.96	0.66	1.39	0.82	0.87	0.59	1.30	0.50
<1 year	3987	18	0.04	11.74	0.64	0.40	1.02	0.06	0.58	0.35	0.95	0.03
≥3 year	3896	31	0.07	11.84	1.08	0.75	1.54	0.69	1.05	0.73	1.51	0.79

Stratified by trial, WHI extension study, and age group.

Base model was adjusted by age.

Multivariable model adjusted for age, history of lupus, history of rheumatoid arthritis, current medical care provider.

Table 4 shows the association of statin use and risk of NHL by overall statin use, statin type, potency, and lipophilicity using time‐dependent models. In the multivariable‐adjusted time‐dependent model, statin use was associated with a borderline significant lower risk of NHL compared to nonusers (HR 0.81, 95% C.I. 0.66–1.00) and in an analysis by statin lipophilicity, there was a suggestion of lower risk of NHL seen with use of lipophilic statins (HR 0.83, 95% C.I. 0.66–1.03). For hydrophilic statins, the HR for NHL was 0.76, 95% C.I. 0.49–1.22).

**Table 4:** Non‐Hodgkin's lymphoma (NHL) HRs by time‐dependent statin use

		Age adjusted				Multivariable adjusted			
	NHL cases	Hazard ratio	Lower CL	Upper CL	*P*‐value	Hazard ratio	Lower CL	Upper CL	*P*‐value
Statin use									
No	660	1.00				1.00			
Yes	53	0.82	0.68	1.00	0.05	0.81	0.66	1.00	0.04
Statin type									
No statin use	660	1.00				1.00			
Atorvastatin calcium	2	0.81	0.60	1.09	0.15	0.84	0.62	1.13	0.24
Fluvastatin sodium	6	0.66	0.31	1.38	0.27	0.58	0.26	1.31	0.19
Lovastatin	17	1.09	0.66	1.79	0.74	1.00	0.59	1.70	0.99
Pravastatin sodium	11	0.79	0.49	1.29	0.35	0.79	0.48	1.30	0.36
Simvastatin	15	0.88	0.62	1.24	0.46	0.85	0.59	1.22	0.37
Lipophilicity									
No statin use	660	1.00				1.00			
Lipophilic statin	40	0.84	0.68	1.04	0.11	0.83	0.66	1.03	0.09
Hydrophilic statin	11	0.77	0.50	1.21	0.26	0.76	0.49	1.22	0.28
Statin potency									
No statin use	660	1.00				1.00			
Low (Lovastatin, Fluvastatin)	23	0.89	0.58	1.35	0.57	0.81	0.52	1.26	0.34
Medium (Pravastatin)	11	0.78	0.48	1.26	0.30	0.77	0.47	1.27	0.31
High (Simvastatin, Atorvastatin)	17	0.83	0.65	1.04	0.11	0.83	0.65	1.06	0.13

Stratified by trial, WHI extension study, and age group.

Base model was adjusted by age.

Multivariable model adjusted for age, current medical care provider, history of lupus, history of rheumatoid arthritis.

Table 5 shows the time‐dependent multivariable‐adjusted relationship between statin use and risk of NHL by histologic subtype. Statin use was associated with a reduced risk of DLBCL (HR 0.62, 95% C.I. 0.42–0.91). This effect was mostly driven by lipophilic statins (HR 0.62, 95% C.I. 0.40–0.96). No association was seen between risk of DLBCL and statin potency or individual statin type. There was no significant relationship between statins and risk of follicular lymphoma (HR 0.96, 95% C.I. 0.64–1.43), marginal zone lymphoma (HR 0.76, 95% C.I. 0.39–1.46), or SLL (HR 0.98, 95% C.I. 0.72–1.34).

**Table 5:** Diffuse large B‐Cell lymphoma (DLBCL) HRs by time‐dependent statin use

		Age adjusted				Multivariable adjusted			
	DLBCL cases	Hazard ratio	Lower CL	Upper CL	*P*‐value	Hazard ratio	Lower CL	Upper CL	*P*‐value
Statin use									
No	215	1.00			0.02	1.00			0.02
Yes	13	0.65	0.45	0.94		0.62	0.42	0.91	
Statin type									
No statin use	215	1.00				1.00			
Atorvastatin	1	0.69	0.40	1.19	0.18	0.69	0.39	1.23	0.21
Fluvastatin	1	0.26	0.04	1.89	0.18	0.000	0.000		0.97
Lovastatin	2	0.59	0.19	1.83	0.36	0.63	0.20	1.98	0.43
Pravastatin	5	0.68	0.28	1.65	0.40	0.58	0.22	1.58	0.29
Simvastatin	4	0.83	0.45	1.52	0.54	0.81	0.43	1.54	0.53
Lipophilicity									
No statin use	215	1.00				1.00			
Lipophilic	8	0.65	0.43	0.97	0.04	0.62	0.40	0.96	0.03
Hydrophilic	5	0.79	0.37	1.67	0.53	0.72	0.32	1.63	0.43
Statin potency									
No statin use	215	1.00				1.00			
Low (Lovastatin, Fluvastatin)	3	0.44	0.16	1.18	0.10	0.35	0.11	1.10	0.07
Medium (Pravastatin)	5	0.67	0.27	1.62	0.37	0.57	0.21	1.54	0.27
High (Simvastatin, Atorvastatin)	5	0.74	0.48	1.12	0.15	0.73	0.47	1.14	0.16

Stratified by trial, WHI extension study, and age group.

Base model was adjusted by age.

Multivariable model adjusted for age, current medical care provider, history of lupus, history of rheumatoid arthritis.

## Discussion

We analyzed the association of statins and risk of NHL over a long period of follow‐up and showed in a time‐dependent analysis that statin use was associated with a borderline lower risk of NHL. Furthermore, we found that statin use is specifically associated with a statistically significant lower risk of DLBCL. The inverse association with statins was restricted to lipophilic statins, but an association with other statins cannot be ruled out. It is also possible that the results may be due to residual confounding.

Table 6 includes an outline of longitudinal studies evaluating the relationship between statin use and NHL risk. Our findings are supported by the results of three other studies demonstrating a reduced risk of NHL among statin users 8, 9, 10; however, two other studies reported no significant impact of statins 11, 12. In an analysis of the Cancer Prevention Study II Nutrition Cohort (CPS‐II), Jacobs et al. demonstrated that statin use for five or more years was associated with a reduction in risk of several cancers including NHL overall (HR = 0.74, 95% CI 0.62–0.89) as well as DLCL (RR = 0.68, 95% CI 0.46–1.00) and marginal zone lymphoma (RR = 0.36, 95% CI 0.15–0.86) 9. In a case–control study nested in a cohort of 547 UK general practices, comprising of 7285 cases of hematologic malignancies (including lymphoma, myeloma, and leukemia), Vinogradova et al. 10 reported that ever use of statins was associated with a reduced risk (HR = 0.78, 95% CI 0.71–0.86). Lastly in the EPI‐LYMPH case–control study comprising 2362 cases of incident lymphomas and 2206 controls, ever use of a statin was associated with a lower risk of lymphoma (OR 0.61, 95% C.I. 0.45–0.84) 8. In contrast, a U.S. case–control study of 4913 cancer cases, of which 144 were NHL cases, did not result in a reduction in risk of lymphoma 12 while a Japanese case–control with 221 cases showed an increased risk of lymphoma associated with statins (OR 2.11, 95% CI 1.20–3.69) 11. Our analysis was only the second cohort study to look at the relationship between statins and lymphoma risk. While in contrast to others, our study only included women by design, we had a larger cohort size than the Jacobs et al. study, however, fewer cases of NHL. In addition, as were others, we were able to analyze the relationship of statin subtype with NHL risk. Our results, however, were comparable to Jacob's et al. showing a lower risk of DLBCL. We, however, did not see a significant reduction in marginal zone lymphoma.

**Table 6:** Longitudinal studies of stains and risk of non‐Hodgkin's lymphoma (NHL)

Ref	Study type	Sample size	Exposure	Outcome	HR, 95% CI
Jacobs 9	Cohort	Cases: 1005 Pop: 133, 255	Current < or > 5 years	NHL DLCL	0.74 (0.62–0.86) 0.68 (0.46–1.00)
Desai	Cohort	Cases: 712 Pop: 161,563	Current < or > 3 years and time dependent	NHL Lipophilic statins DLCL	0.81 (0.66–0.99) 0.82 (0.66–1.03) 0.61 (0.41–0.91)
Vingradova 10	1 Nested Case–Control	Cases of heme cancers: 7185 Controls: 29,162	Statin yes vs. no	Hematologic Malignancies (including leukemia, myeloma, and NHL)	0.61 (0.41–0.89)
Epilymph study 11	Case–Control	Cases‐2362 Controls: 2206	Ever use of statins	NHL (B, T, and NK)	0.61 (0.41–0.89)

Results from a number of preclinical studies suggest that our current observation is biologically plausible. Statins have been previously shown to have chemopreventive effects for a large number of cancer types based on their downstream molecular effects 21, 22. Reports examining the in vitro impact of statins have shown that statins possess antiproliferative, apoptotic, and anti‐invasive properties resulting from their ability to target multiple signaling pathways within malignant cells 21, 22, 23, 24, 25, 26, 27, 28, 29. Statins have been shown to reduce the farnesylation of Ras needed for its attachment to the cellular membrane 23, and Ras is involved in many intracellular pathways and has been shown to increase gene transcription and proliferation via MEK and PI3K/Akt signaling 21.

Statins have also been shown to inhibit the production of FPP and GPPP, two key downstream products involved in posttranslational modification of many proteins including geranylgeranylation of Rho proteins (Rho GTPases) 30. In turn, these gene products regulate Rho kinases, which are involved in various cellular functions including gene expression, actin cytoskeleton migration, adhesion, and contractility of cells 31. Thus, by inhibiting the production of GGPP, statins may have antiproliferative and anti‐invasive properties. Statins have also been implicated in G1‐S arrest 32. Lastly, studies using transgenic mouse models have demonstrated that statins possess proapoptotic and antitumor activity against lymphoma 5. Data from Ajith et al. indicate that statin‐induced apoptosis depends on their ability to inhibit lipid peroxidation and depleted key geranylgeranylated proteins. Shachaf et al. have described a transgenic mouse model in which atorvastatin reverses the development of MYC‐induced lymphomas in a dose‐dependent fashion 6. In this latter model, atorvastatin was found to delay the onset of lymphoma, helping to purge neoplastic cells from the bone marrow. These effects were negated if Ras was constitutively activated. The specific pathway by which statins exert this effect is by inactivating MYC following disruption of upstream Ras/ERK ½ pathways.

Another mechanism by which statins may impact risk of lymphoma is through inhibition of histone deacetylases (HDACs). It has been recently shown that the carboxylic moiety of lovastatin can bind and chelate the catalytic site of HDAC2, leading to increased p21 expression and inhibition of tumor cell growth 33. HDAC inhibition has been targeted in a number of hematologic malignancies including lymphoma 34. Lastly, the anti‐inflammatory effect of statins may be an important mechanism of reduction in risk of lymphoma as chronic inflammation is a possible risk factor for lymphoma 7.

Our observation that statins differentially impact lymphoma risk based on their biologic properties is also consistent with the exiting medical literature [21, 35]. Statins are classified according to their solubility in octanol (lipophilicity) and water (hydrophilicity) 16. Lipophilic statins (lovastatin, simvastatin fluvastatin, and atorvastatin) penetrate the plasma member while hydrophilic statins (pravastatin) do not. A number of in vitro studies have shown that lipophilic statins possess the most robust anticancer properties, consistent with the observations reported above.

The strengths of our analysis include the prospective design, the large diverse population with detailed demographic characterization, adjudicated cancer diagnosis by central review, serial update of statin use, and long follow‐up period. The comprehensive data collection in the WHI also allows for a detailed adjustment for confounding variables. Limitations include the observational nature and the relatively low prevalence of statin use at baseline among WHI subjects. Despite these limitations, we were able to capture updated information on statin use using a time‐dependent analysis. Another limitation is the lack of data documenting medication compliance and HIV status, another risk factor for NHL. It is currently thought that the prevalence of HIV in the WHI cohort of postmenopausal women is low. We also did not have dose information on statins and were not able to analyze relationship of dosing or duration of use in the time‐dependent analysis. Our results suggesting a relationship between lipophilic statins and NHL may be particularly important for high‐risk populations such as those with autoimmune conditions. Our findings showing a specific impact on DLBCL have implications for future studies of the impact of statins on risk of recurrent disease.

## Conclusion

Statin use may be associated with a lower risk of NHL in women particularly DLBCL. These results should be evaluated in other large datasets, particularly meta‐analyses of trial data, with a particular emphasis on specific type of NHL.

## Conflicts of Interests

None of the authors have any conflicts with regard to this manuscript.
